# Molecular mechanism of the anti-gastric cancer activity of 1,2,3,6-tetra-O-galloyl-β-D-glucose isolated from *Trapa bispinosa* Roxb. shell in vitro

**DOI:** 10.1371/journal.pone.0269013

**Published:** 2022-06-02

**Authors:** Limei Wang, Dongjie Yin, Yanhui Fan, Ting Min, Yang Yi, Hongxun Wang

**Affiliations:** 1 College of Life Science and Technology, Wuhan Polytechnic University, Wuhan, China; 2 College of Food Science and Engineering, Wuhan Polytechnic University, Wuhan, China; Huazhong University of Science and Technology, CHINA

## Abstract

*Trapa bispinosa* Roxb. is a traditional Chinese food which is well known for its medicinal properties. The shell of *Trapa bispinosa* has anticancer activity, maybe due to its high content of polyphenols. There are few studies on the chemical composition of *Trapa bispinosa* shells, then we isolated the active components from *Trapa bispinosa* shell and clarified the mechanism of its anticancer activity. One monomer compound was separated from the ethanol extract of the *Trapa bispinosa* shell by fractional extraction, silica gel, Sephadex LH-20 gel column chromatography and liquid phase separation. The structure, identified by NMR was 1,2,3,6-tetra-O-galloyl-β-D-glucose. The results of the CCK-8 assay showed that 1,2,3,6-tetra-O-galloyl-β-D-glucose could significantly inhibit the proliferation of gastric cancer SGC7901 cells, and the effect was close to that of 5-fluorouracil. Here, 1,2,3,6-tetra-O-galloyl-β-D-glucose could affect the cell cycle of SGC7901 cells. At the dose of 200 μg/mL and an incubation time of 48 h, SGC7901 cells remained in the G1 phase, apoptosis occurred, the intracellular calcium ion concentration increased and the mitochondrial membrane potential decreased. Transcriptome sequencing analysis showed that the differentially expressed genes were mainly enriched in the P53 signalling pathway associated with apoptosis. The results of qPCR and Western blot showed that 1,2,3,6-tetra-O-galloyl-β-D-glucose could induce apoptosis of SGC7901 cells by up-regulating the expression levels of *P21*, *PUMA*, *PERP* and *IGF-BP3* genes, down-regulating the *CyclinD* gene, increasing the expression levels of cytochrome C, caspase-3, caspase-9 protein and decreasing that of the protein BCL-2.

## 1. Introduction

*Trapa bispinosa* Roxb. (TbR), which commonly known as Water chestnut or Shuili and Shajiao in China, is an annual aquatic herb belonging to the family Trapaceae. It is native to temperate and tropical regions of Southeast Europe, Asia and Africa and is commercially cultivated in China, India, Japan and Southeast Asia [1, 2] and grows in shallow water fields, lakes and irrigation ponds. The current planting area is about 40,000 hectares, with an annual output of about 240,000 tons, mainly concentrated in dense lake areas [3]. Based on previous studies, *Trapa bispinosa* is rich in active ingredients, which occur in different plant parts. Qualitative analysis has shown that *Trapa bispinosa* fruits contain carbohydrates, reducing sugar, starch, protein and flavonoid [4], and the methanol extract has anti-bacterial [5] and immunomodulatory activity [6]. Leaves of *Trapa bispinosa* contain carbohydrates, phenols, saponins, carboxylic acid, fixed oils and fats in petroleum ether extract [7] and have antioxidant and antiproliferative activities [8].

The dried fruit shell of *Trapa bispinosa* is often used in tea and other herbal medicines in India and Japan. As a by-product of food processing, large amounts of TbR shells are usually discarded as waste each year in China. However, extracts of TbR shells have beneficial properties, such as antioxidant activity [5, 9], antibacterial activity [10], cytotoxic activity [11], antidiabetic activity [12] and inhibition of glycometabolism [13]. The literature indicates that the extract of TbR shells has anti-tumour activity. Ethanol extract of TbR shells has a high inhibitory effect on the proliferation of liver cancer SMMC-7721 cells [14] and lung cancer A549 cells [15]. Some studies have shown that the TbR shell is rich in polyphenols, flavonoids and alkaloids [16–18], with polyphenolic compounds being the most active ones [19–21]. Niu Fenglan et al. have isolated a polyphenol compound from the ethyl acetate extract of *Toxoplasma japonica* and identified it as a dimer of 3,4,5-trihydroxybenzoic acid, inhibiting the activity of gastric cancer cells [22]. Similarly, Lin Qiusheng et al. have systematically studied the activity of two monomeric compounds (gallic acid and gallic acid dimer) in the ethyl acetate extract and their inhibition of the activity of gastric cancer cells [23]. Other studies have shown that polyphenolic compounds have anti-tumour effects [24–26], but the underlying mechanisms are complex. Polyphenols could directly kill tumour cells [27], blocking the cell cycle [28] and inhibiting tumour growth by improving the body’s immunity and antioxidant activity [29].

In our preliminary research, we showed that graded extracts of *Trapa bispinosa* shell could inhibit gastric cancer SGC-7901 cell proliferation, causing apoptosis [30]. These studies were performed using concentrated crude extracts or simple purification of TbR shells. However, the main active compounds in these extracts have rarely been determined, and the underlying molecular mechanisms need to be further elucidated. In this context, the present study has been designed to isolate the monomeric compounds with tumour-suppressing activity and to identify the chemical structure by NMR. One new compound, 1,2,3,6-tetra-O-galloyl-β-D-glucose, was isolated, and the structure was verified through analysis of spectroscopic data (1H NMR, 13C NMR). The inhibitory effects against human gastric cells and the molecular mechanism were characterised.

## 2. Materials and methods

### 2.1 Chemicals

DMSO was obtained from Hubei Baisi Biotechnology Co., Ltd. Foetal bovine serum (FBS), penicillin and streptomycin were purchased from Hangzhou Sijiqing Bioengineering Materials Co., Ltd., and 5-fluorouracil (5-Fu) was obtained from Sigma. The DMEM medium was purchased from Hyclone, while the cell counting kit (CCK-8) was obtained from Japan Tongren Reagent Co., Ltd. The T25 flasks, 96-well plates and 6-well plates were purchased Coming, USA. Trypsin was obtained from the Hangzhou Gino Biomedical Technology Company, and anhydrous ethanol, n-butanol, chloroform and methanol from Sinopharm Chemical Reagent Co., Ltd. The 40–60 μm silica gel was obtained from Qingdao Ocean Chemical. The real-time PCR kit, Sephadex LH-20, was obtained from BioRad, the total RNA extraction kit from Omega and the reverse transcription kit from Takara. The cell cycle detection kit, the mitochondrial membrane potential detection kit and the intracellular calcium ion concentration detection kit were obtained from Solite. The apoptosis assay kit was purchased from BestBio.

### 2.2 Materials

The human gastric cancer cell line SGC7901, DMSO, was obtained from Hubei Baisi Biotechnology Co., Ltd. *Trapa bispinosa Roxb*. shells were collected in September 2018 in Wuhan, Hubei province, P.R. China, and identified by Associate Professor Wanyong Zeng. The shells were oven-dried for 3 days at 40°C and stored at 4°C.

### 2.3 Preparation of plant extracts

The dried *Trapa bispinosa* shells (200 g) were crushed and extracted with 10 equivalent volumes of 70% ethanol. The obtained extracts were filtrated, concentrated in a vacuum evaporator in a water bath and then further extracted with petroleum ether, ethyl acetate and n-butanol. Subsequently, they were separated by column chromatography on a column containing 100 g of silica gel (60 mesh) and eluted with a mixture of ethyl acetate and petroleum ether (3:6 v/v), followed by elution with a mixture of chloroform and methanol (2:8 v/v) to collect the A component. Fraction A (8 g) was processed by Sephadex LH-20 gel column chromatography with a gradient elution of methanol: H_2_O (30:70 to 100:0). The collected four components (B1, B2, B3, B4), in particular, the B3 component, had highest inhibiting effect on SGC7901 tumour cell proliferation. We then obtained the monomeric compounds (1.2 g) from the high-performance liquid phase. A small amount of monomeric compounds were dissolved in methanol for chromatography and analyzed by LC-MS (Agilent,6410, CA, USA). The m/z scanning range of the mass-to-charge ratio was 0~1000. Subsequently, the monomeric compounds were elucidated by the 1H, 13C and DEPT NMR techniques, using a 600 MHz Agilent NMR system (Agilent, Santa Clara, CA, USA).

### 2.4 Cell culture and treatments

Human gastric cancer SGC7901 cells were cultured in DMEM medium supplemented with 5% heat-inactivated FBS, 100 U mL^−1^ of penicillin and 0.1 mg mL^−1^ of streptomycin and grown at 37°C with 5% CO_2_ and 95% air. Cells were cultured every 3–4 days, and DMSO was used as the vehicle for treatment delivery to the cells; the final concentration of DMSO in the culture medium was 0.1% v/v for all experiments.

### 2.5 Cell viability assay

To determine the effects of the compounds on short-term proliferation, CCK-8 assays were performed. The SGC7901 cells were seeded at 5 × 10^3^ cells per well in a 96-well culture plate and grown at 37°C in a 5% CO_2_ atmosphere for 24 h. Subsequently, the cells were incubated with various concentrations of isolated compounds and 5-fluorouracil (12.5, 25, 50, 100, 200 μg/mL, dissolved in dimethyl sulfoxide (DMSO) for 24 and 48 h, respectively. After this, the cells were incubated with a 10 μL CCK-8 reagent per well for 3 h. The absorbance of the formazan products was measured at 450 nm by using a microplate reader [31]. The cell inhibition rate was calculated by dividing the number of viable cells in the compound-treated groups by that in the control group, using the following equation:

$$\text{Cell}\;\text{inhibition}\;\text{rate}\;\left(\%\right)=\left[1-\left({\text{OD}}_{\text{test}\;\text{group}}-{\text{OD}}_{\text{blank}\;\text{group}}\right)/\left({\text{OD}}_{\text{control}\;\text{group}}-{\text{OD}}_{\text{blank}\;\text{group}}\right)\right]\times 100.$$

The half maximal inhibitory concentration (IC_50_) was then calculated and considered as the concentration accounting for 50% of the viability.

### 2.6 Cell cycle analysis

Cell cycle analysis was performed using PI staining to measure the DNA content and the distribution of cells in various cell cycle stages. The SGC-7901 cells (1 × 10^6^ cells/well) were seeded in a 6-well plate and treated with isolated compounds (12.5, 25, 50, 100, 200 μg/mL) for 48 h. The cells were then harvested, washed with PBS, fixed in 70% ethanol and stored at -20°C for 4 h. After incubation, cells were re-suspended in PI staining solution (50 μg/mL of PI, 0.1% (v/v) of Triton X-100 and 100 μg/mL of RNase A) and further incubated for 30 min at 37°C in the dark [32]. The total DNA content was measured by CytoFLEX flow cytometry (Beckman Coulter, Miami, FL, USA).

### 2.7 Annexin-V FITC/PI assay

Apoptosis was measured using a commercial Annexin-V FITC/PI kit according to the manufacturer’s instructions. The SGC-7901 cells were harvested and resuspended in binding buffer (1.0 × 10^5^ cells/mL). Subsequently, the cells were stained with 10 μL of annexin-V-FITC and 5 μL of propidium iodide (PI) for 15 min at room temperature in the dark and measured by CytoFLEX flow cytometry. The CytoFLEX Flow cytometric data were collected and analysed using the FlowJo 10 software (TreeStar, Ashland, OR, USA).

### 2.8 Assessment of the mitochondrial membrane potential (MMP ΔΨm)

We used JC-1 staining to determine the MMP loss in SGC-7901 cells after being treated with the isolated compounds. Cells (5 × 10^5^ cells/well) seeded in a six-well plate were treated with the compounds, followed by incubation for 48 hours. Cells were then stained with 1 mL of JC-1 stain and again incubated for 30 min [33]. The excess dye was removed by washing with JC-1 buffer, and the cells were then measured via CytoFLEX flow cytometry (Beckman Coulter, Miami, FL, USA).

### 2.9 Intracellular calcium determination

Cells (5 × 10^5^ cells/well) seeded in a six-well plate were treated with the compounds, followed by incubation for 48 h. Cells were harvested, washed with Hank’s balanced salt solution (HBSS, BI) and resuspended in 1 ml Fura 2-AM working solution (5 μM) at a concentration of 1 × 10^6^ cells/mL. Cells were incubated for 20 min, and the Fura 2-AM working solution was then removed. Cells were washed once with HBSS and incubated for 1 h in HBSS, followed by measuring via CytoFLEX flow cytometry (TreeStar, Ashland, OR, USA).

### 2.10 Transcriptomic data analysis, RNA extraction and quantitative RT-PCR

A total of six samples of SGC7901 cells were prepared. Three control groups were treated with 50 μg/mL of 1,2,3,6-tetra-O-galloyl-β-D-glucose for 24 h, and finally, all samples were extracted via the TRIZOL method. Transcriptome sequencing was performed using the BGISEQ-500 platform.

Raw reads were first discarded if they contained more than 10% N bases, adaptor or low-quality reads using SOAPnuke (version 1.5.2). The clean reads were aligned to the reference genome by HISAT (version 2.0.4), and FPKM values (FPKM represents fragments per kilobase and per million) were calculated using RSEM (version 1.2.12). Differentially expressed genes (DEGs) between the control and treatment groups were analysed using DEseq2. Functional enrichment analysis of DEGs in gene ontology (GO) and the KEGG pathway database were applied using phyper in the R package.

The RNA was isolated from cells using TRIZOL (Takara, Dalian, China), and RNA quantity and quality were determined using the Nanodrop ND-1000 spectrophotometer (Thermo Fisher Scientific, Waltham, MA, USA) at a wavelength of 260/280 nm. The cDNA was generated from 5 ug of total RNA, using SuperScript IV reverse transcriptase. The 20-uL amplification reaction consisted of iTaq Universal SYBR ^®^ Green Supermix (BioRad, Hercules, CA, USA) with 300 nM of both reverse and forward primers (Table 1). All reactions were performed on a CFX96 real-time PCR System (BioRad, Hercules, CA, USA). The thermal cycling conditions were 50°C for 2 min and 95°C for 3 min, followed by 40 repeats at 95°C for 15s, 60°C for 30 s and 72°C for 30 s. To control for variations in the reactions, the amount of target mRNA was normalised to invariable control gene Beta-Actin expression. The comparative threshold cycle (Ct) method was used to determine the amount of target gene normalised to tuba4a and relative to a calibrator 2 ^−ΔΔCt^. The purity of the PCR products was verified by melting curves and gel electrophoresis.

**Table 1:** PCR amplified primer sequence.

Gene	Forward primer (5’→3’)	Reverse primer (5’→3’)
*PUMA*	GAGGAGGAACAGTGGGCC	GGAGTCCCATGATGAGATTGT
*P21*	GACACCACTGGAGGGTGACT	CAGGTCCACATGGTCTTCCT
*CyclinD*	AACTACCTGGACCGCTTCCT	CCACTTGAGCTTGTTCACCA
*IGF-BP3*	CCTGCCGTAGAGAAATGGAA	AGGCTGCCCATACTTATCCA
*PERP*	TGCCATCATTCTCATTGCAT	AACCCCAGTTGAACTCATGG
*β-Actin*	CATCCGCAAAGACCTGTACG	CCTGCTTGCTGATCCACATC

### 2.11 Western blot analysis

Total cytoplasmic proteins from each sample were extracted using a microplate BCA protein assay kit (Pierce, Rockford, IL, USA). Cell protein extracts were mixed with sample buffer (62.5 mmol/L Tris-HCl pH 6.8 (BioRad), 25% glycerol, 2% SDS, 350 mmol/L DTT and 0.01% bromophenol blue) at a ratio of 1:1 (v/v). The samples were then incubated in boiling water for 5 min, and aliquots of the samples (30 mg of protein) were separated by electrophoresis in 12.5% SDS-PAGE gels at a constant voltage (120 V) and then transferred to polyvinylidene difluoride membranes (Millipore, USA). The membranes were blocked with 5% milk and incubated overnight at 4°C with the following primary antibodies: anti-pIRS1(Ser307) (1:1,000; AI623-1, Beyotime, China) and anti-IRS1 (1:1,000; 2382, CST, USA), while beta-actin was selected as internal control. The optical densities of the bands of interest were analysed quantitatively using the Quantity One software (Bio-Rad, USA).

### 2.12 Statistical analysis

All data are presented as the mean ± SD values, with normal distribution. Differences among the groups were determined by one-way ANOVA, followed by Turkey’s test for equality of variances, using Graphpad Prism (version 7.0). Differences were considered statistically significant at *P* < 0.05.

## 3. Results

### 3.1 Identification of the isolated components of the TbR shell

**Fig 1:**
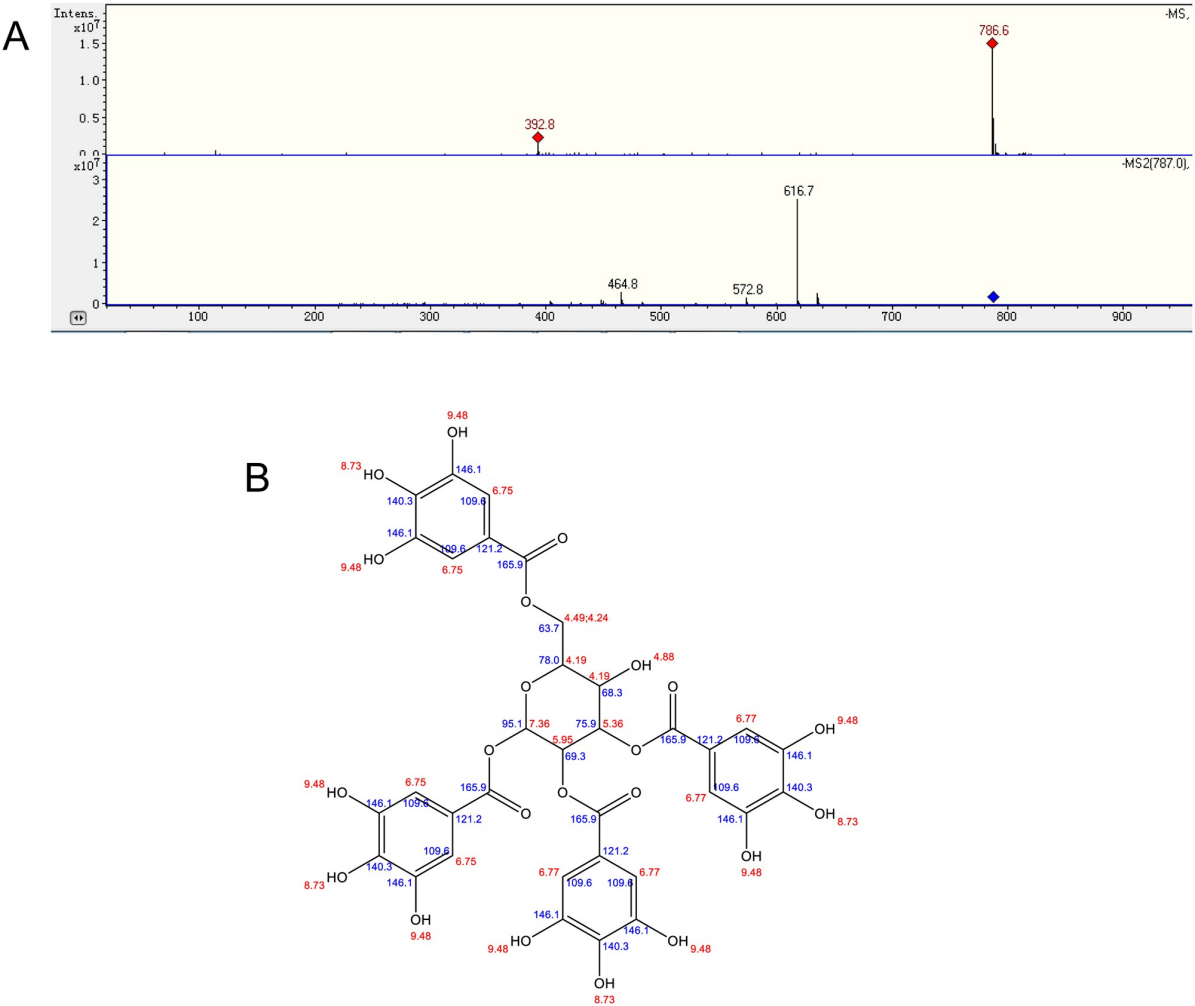
Structural formula of the compound separated from the ethanol extract of the *Trapa bispinosa* shell. A. LC/MS analysis of the monomer compound. B. Structural formula of monomer compound.

We isolated one compound from the TbR shell. It was a yellow powder, ESI-MS m/z:787[M-H]^-^ (Fig 1A), 1H-NMR(600MHz,MEOD)δ 6.92(2H,s,Ar6-H2,6), 6.91(2H,s,Ar1-H2,6), 6.88(2H,s,Ar3-H2,6), 6.81(2H,s,Ar2-H2,6), 6.13(1H,d,J = 8.4Hz,H-1), 5.52(1H,dd,J = 9.6,9.5Hz,H-2), 5.27(1H,dd,J = 8.4,7.9Hz,H-3), 4.44(1H,d,J = 9.4Hz,H-6a), 4.40(1H,dd,J = 7.8,4.4Hz,H-6b), 4.08(1H,d,J = 7.6Hz,H-5), 3.81(1H,d,J = 4.8Hz,H-4). ^13^C NMR(151MHz,MEOD)δ 165.6(6-Gall-C 7), 165. 0(3-Gall-C7), 164. 6(2-Gall-C7), 163.9(1-Gall-C7), 145.5(1,3,6-Gall-C3,5), 145.3(2-Gall-C3, 5), 139. 4(1-Gall-C4), 138.9(2-Gall-C4), 138.5(6-Gall-C4), 138.5(3-Gall-C4), 119.9(6-Gall-C1), 118.9(3-Gall-C1), 118.1(2-Gall-C1), 117.5(1-Gall-C1), 108.9(1-Gall-C2,6), 108.7(2, 3-Gall-C2,6), 108.6(6-Gall-C2,6), 92.0(Glc-C1), 76.3(Glc-C3),74.8(Glc-C5), 70.6(Glc-C2), 67.4(Glc-C4), 62.8(Glc-C6) (Fig 1B). The structure was 1,2,3,6-tetra-O-galloyl-β-D-glucose (TGG), determined via analysis of the NMR spectra and by comparing the spectral data with data found in the literature [34, 35].

### 3.2 1,2,3,6-tetra-O-galloyl-β-D-glucose suppress SGC7901 cell proliferation and arrest the cell cycles

Based on Table 2, 1,2,3,6-tetra-O-galloyl-β-D-glucose has a certain inhibitory effect on the proliferation of SGC7901 cells. As the concentration increased, the inhibition rate also increased. Compared with the 5-FU inhibition effect, when the sample concentration was 12.5 μg/mL, the proliferation inhibition rate of 5-FU on SGC7901 was higher than that of 1,2,3,6-tetra-O-galloyl-β-D-glucose. When the concentration was 200 μg/mL, the inhibition rate of l,2,3,6-tetra-O-galloyl-β-D-glucose on SGC7901 was over 70% and equivalent to that of 5-FU. The semi-inhibitory concentration (IC_50_ value) was calculated based on the strength of the inhibition rate (Table 3). The IC_50_ values of the proliferation inhibition rate of l,2,3,6-tetragalloyl-β-D-glucose on SGC7901 were 59.15 and 36.23 μg/mL, respectively. The inhibitory effect of 1,2,3,6-tetra-O-galloyl-β-D-glucose on SGC7901 cells was not as good as that of 5-FU.

**Table 2:** l,2,3,6-tetra-O-galloyl-β-D-glucose inhibits tumor cell proliferation activity.

Sample	Time	SGC7901cell growth inhibition rate (%)				
		12.5μg/mL	25μg/mL	50μg/mL	100μg/mL	200μg/mL
l, 2, 3, 6-tetra-O-galloyl-β-D-glucose	24h	6.98±4.66	29.39±1.46	51.51±1.46	65.58±3.50	75.00±4.73
	48h	24.08±8.90	43.37±4.21	61.15±3.08	73.86±3.14	80.27±1.15
5-Fu	24h	59.32±2.93	66.00±3.13	67.10±4.14	60.83±3.33	71.54±2.94
	48h	69.08±1.90	70.38±4.80	81.73±4.50	90.04±2.50	94.88±1.30

**Table 3:** IC_50_ values of l,2,3,6-tetra-O-galloyl-β-D-glucose on SGC7901 cells.

Time	IC_50_ (μg/mL)	
	L,2,3,6-tetra-O-galloyl-β-D-glucose	5-Fu
24h	59.15	9.12
48h	36.23	4.06

**Fig 2:**
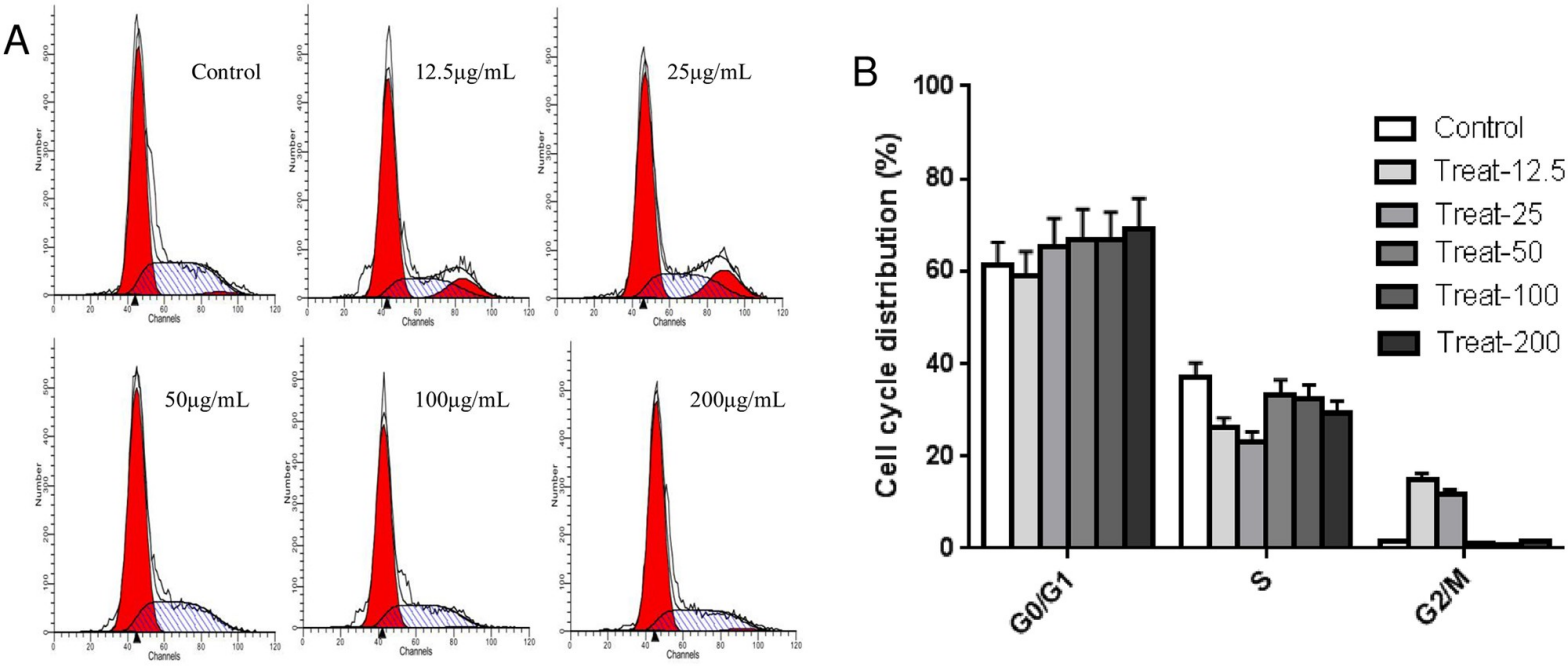
Evaluation of cell cycle in SGC-7901 cells after l,2,3,6-tetra-O-galyl-β-D-glucose treatment. A. Intracellular the fluorescence intensity in SGC-7901 cells cultured in the presence of 1,2,3,6-tetra-O-galloyl-β-D-glucose (0, 12.5,25, 50, 100 and 200 μg/mL) for 48 h. B. Average intensity of fluorescence in SGC-7901cells.

**Fig 3:**
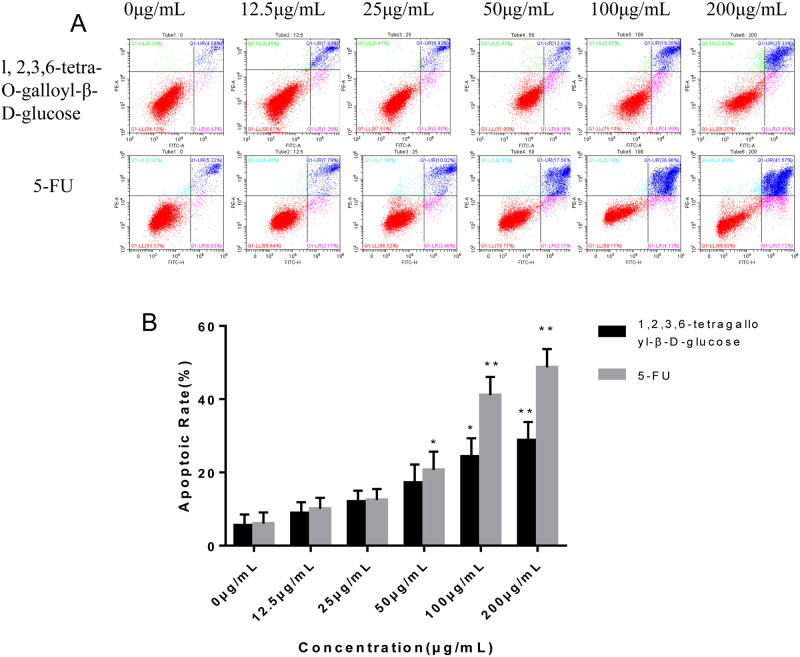
Effects of different concentrations of 1,2,3,6-tetra-O-galloyl-β-D-glucose treatment on apoptosis of SGC7901 cells compared with 5-Fu. A. FITC-Annexin V/PI double-staining flow cytometry for detecting apoptosis rate of SGC7901 cells after 1,2,3,6-tetra-O-galloyl-β-D-glucose and 5-FU treatment (0, 12.5,25, 50, 100 and 200 μg/mL) for 48 h, respectively. B. Average Apoptosis rate in SGC-7901cells.

As shown in Fig 2, 1,2,3,6-tetra-O-galloyl-β-D-glucose was used to incubate SGC-7901 cells for 48 h. At a l,2,3,6-tetra-O-galloyl-β-D-glucose dose of 12.5 and 25 μg/mL, the S phase cells decreased significantly (*P* < 0.05), while the G2/M phase cells increased significantly (*P* < 0.05) compared with the control group. With increased drug concentration, the S phase and G2/M phase cells remained basically unchanged compared with the control group, and G0/G1 phase cells slightly increased. The results showed that 1,2,3,6-tetra-O-galloyl-β-D-glucose could slightly arrest SGC7901 cells in the G1 phase, without a significant difference at high concentrations (100 and 200 μg/mL).

### 3.3 1,2,3,6-tetra-O-galloyl-β-D-glucoseinduce SGC7901 cell apoptosis

As shown in Fig 3, human gastric cancer cells SGC7901 were treated with different concentrations of 1,2,3,6-tetra-O-galloyl-β-D-glucose for 48 h. This substance increases the number of apoptotic cells in a concentration-dependent manner. The apoptotic rates were 8.89, 12.01, 17.18, 24.34 and 28.78% at doses of 12.5, 25, 50, 100 and 200 μg/mL, respectively. The apoptotic rate was significantly different at high concentrations (100 and 200 μg/mL) (*P* < 0.05) compared with the control group. The early apoptosis rate of cells was 3.45% at the concentration of 200 μg/mL, while the late rate was 25.33%. Based on Fig 3, the apoptosis rate increases with increasing 5-FU concentrations. The apoptotic rates were 9.96, 12.48, 20.67, 41.09 and 48.96% at doses of 12.5, 25, 50, 100 and 200 μg/mL, respectively. The apoptosis rate was extremely significant (*P* < 0.01) at doses of 100 and 200 μg/mL compared with the control group. The early apoptotic rate of cells was 7.12%, while the late apoptotic rate was 41.57% at 200 μg/mL. Based on the above results, l, 2,3,6-tetra-O-galloyl-β-D-glucose could induce apoptosis of human gastric cancer SGC7901 cells *in vitro*, and the effect was 60% of that of the positive drug 5-FU.

### 3.4 Effect of 1,2,3,6-tetra-O-galloyl-β-D-glucose on mitochondrial membrane potential

**Fig 4:**
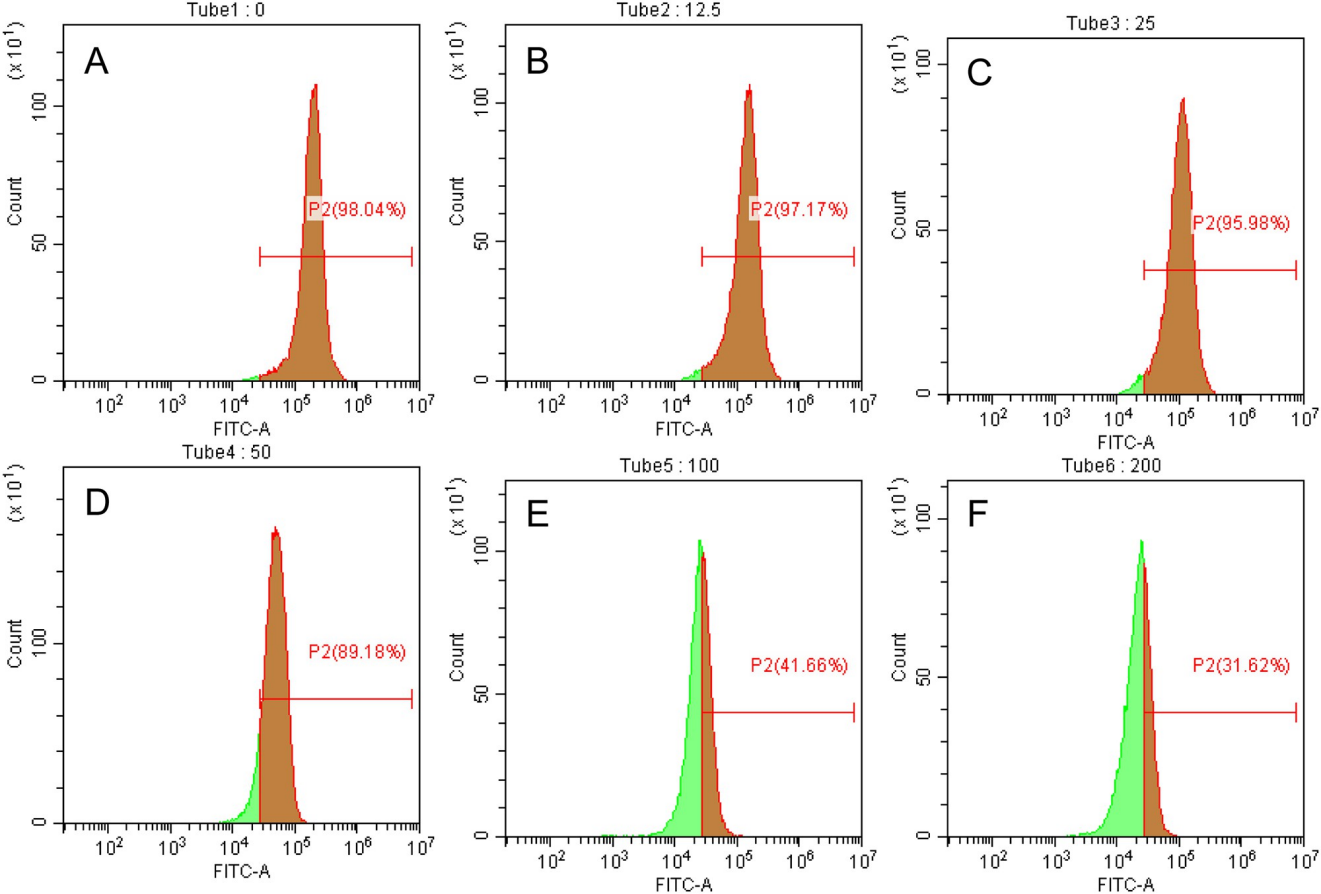
Effect of l,2,3,6-tetra-O-galyl-β-D-glucose on mitochondrial membrane potential (Δψm) of SGC7901 cells. SGC7901 cells were incubated with different concentrationsof 1,2,3,6-tetra-O-galloyl-β-D-glucose for 48 h. The Δψm was evaluated using JC-1 in treated cells. A.0μg/mL, B.12.5 μg/mL C.25μg/mL, D.50μg/mL, E. 100μg/mL, F.200 μg/mL.

The SGC7901 cells were treated with different concentrations of 1,2,3,6-tetra-O-galloyl-β-D-glucose for 48 h, and the mitochondrial membrane potential was measured by JC-1 staining. The mitochondrial membrane potential of gastric cancer cells SGC7901 was decreased after treatment with 1,2,3,6-tetra-O-galloyl-β-D-glucose (Fig 4). Compared with the control group, 1,2,3,6-tetra-O-garoyl-β-D-glucose only slightly affected the membrane potential of gastric cancer cells at low concentrations (12.5–50 μg/mL). When the drug concentration reached 100 μg/mL, the mitochondrial membrane potential decrease rate was 56.38%, which was significantly different from that of the control group (*P* < 0.05). The highest concentration (200 μg/mL) of 1,2,3,6-tetra-O-galloyl-β-D-glucose reduced the mitochondrial membrane potential of SGC7901 cells by up to 66.42%. These experimental results demonstrate that 1,2,3,6-tetra-O-galloyl-β-D-glucose induced apoptosis of SGC7901 cells by decreasing the mitochondrial membrane potential and increasing the mitochondrial membrane permeability.

### 3.5 Effect of 1,2,3,6-tetra-O-galloyl-β-D-glucose on intracellular calcium

**Fig 5:**
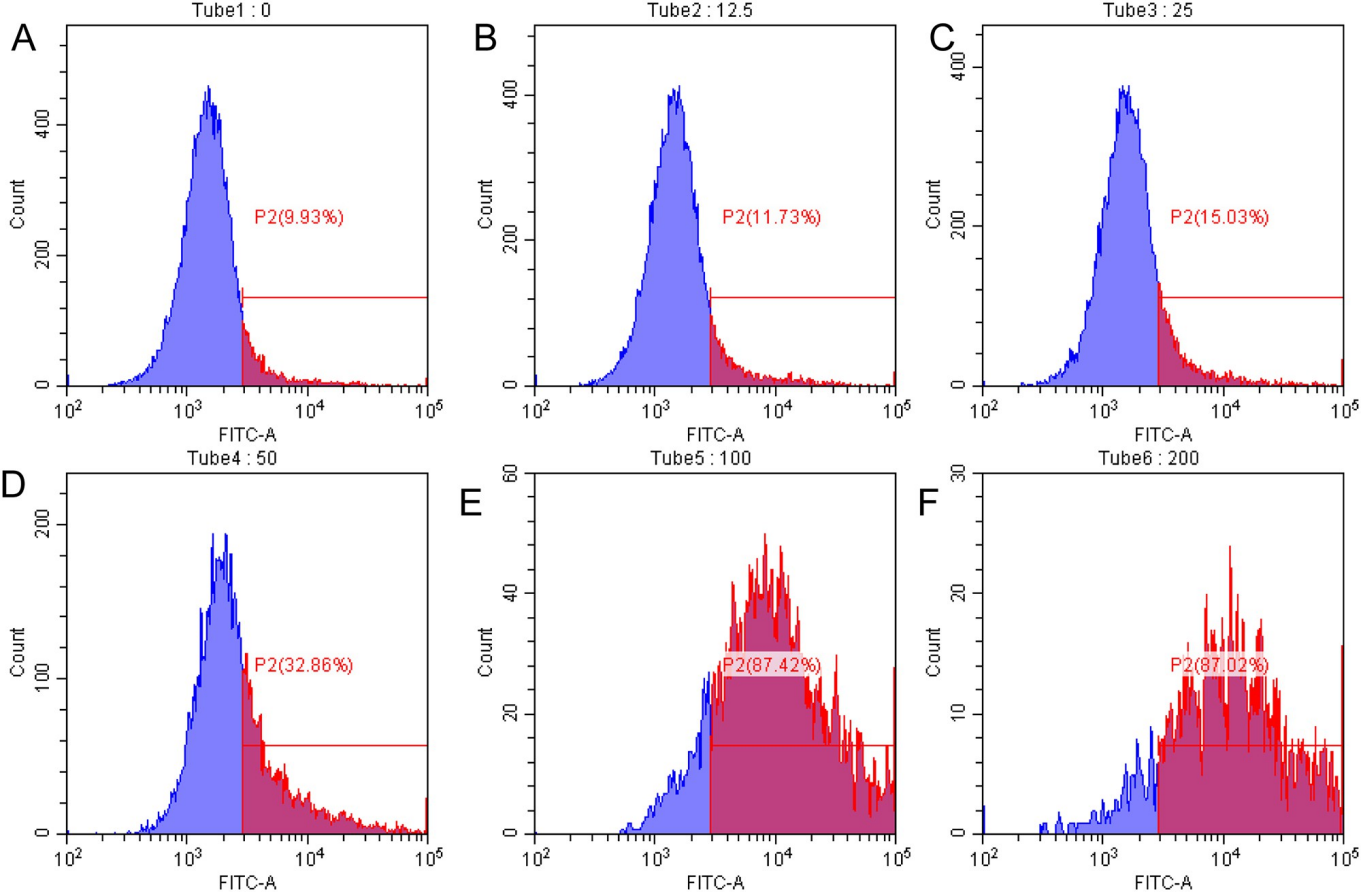
Effect of l,2,3,6-tetra-O-galloyl-β-D-glucose on intracellular calcium concentration in SGC7901 cells. SGC7901 cells were incubated with different concentrations of 1,2,3,6-tetra-O-galloyl-β-D-glucose for 48 h. Fura 2-AM fluorescence photometry was used to measure [Ca^2+^]i. A.0μg/mL, B.12.5 μg/mL C.25μg/mL, D.50μg/mL, E.100μg/mL, F.200 μg/mL.

The concentration of Ca^2+^ in the cytoplasm of SGC7901 cells was measured by flow cytometry after adding different concentrations of 1,2,3,6-tetra-O-galloyl-β-D-glucose. The Fluo-3-AM decomposed into Fluo-3 in the cells, which could bound to free Ca^2+^, and the emitted fluorescence could be captured by flow cytometry. The total fluorescence intensity of each group of cells was shown in the spectrum. The Count axis shows the amount of cells loaded, and the FITC-A measured the fluorescence intensity of the cells. Fig 5 shows that the number of cells captured by the cytometer and the fluorescence intensity of SGC7901 cells increased with increasing concentrations of 1,2,3,6-tetra-O-galloyl-β-D-glucose. Compared with the control group, the intracellular calcium concentration did not change significantly when the drug concentration was 12.5–50 μg/mL. However, the intracellular calcium concentration increased significantly (*P* < 0.05) when the concentration of l,2,3,6-tetra-O-galloyl-β-D-glucose was 100–200 μg/mL and reached 77% at a concentration of 200 μg/mL. Thus, l,2,3,6-tetra-O-galloyl-β-D-glucose-mediated apoptosis of SGC7901 cells is closely related to the increase in intracellular free calcium ion concentration.

### 3.6 Comparative transcriptomic study and qPCR validation between the control and the treatment group

**Fig 6:**
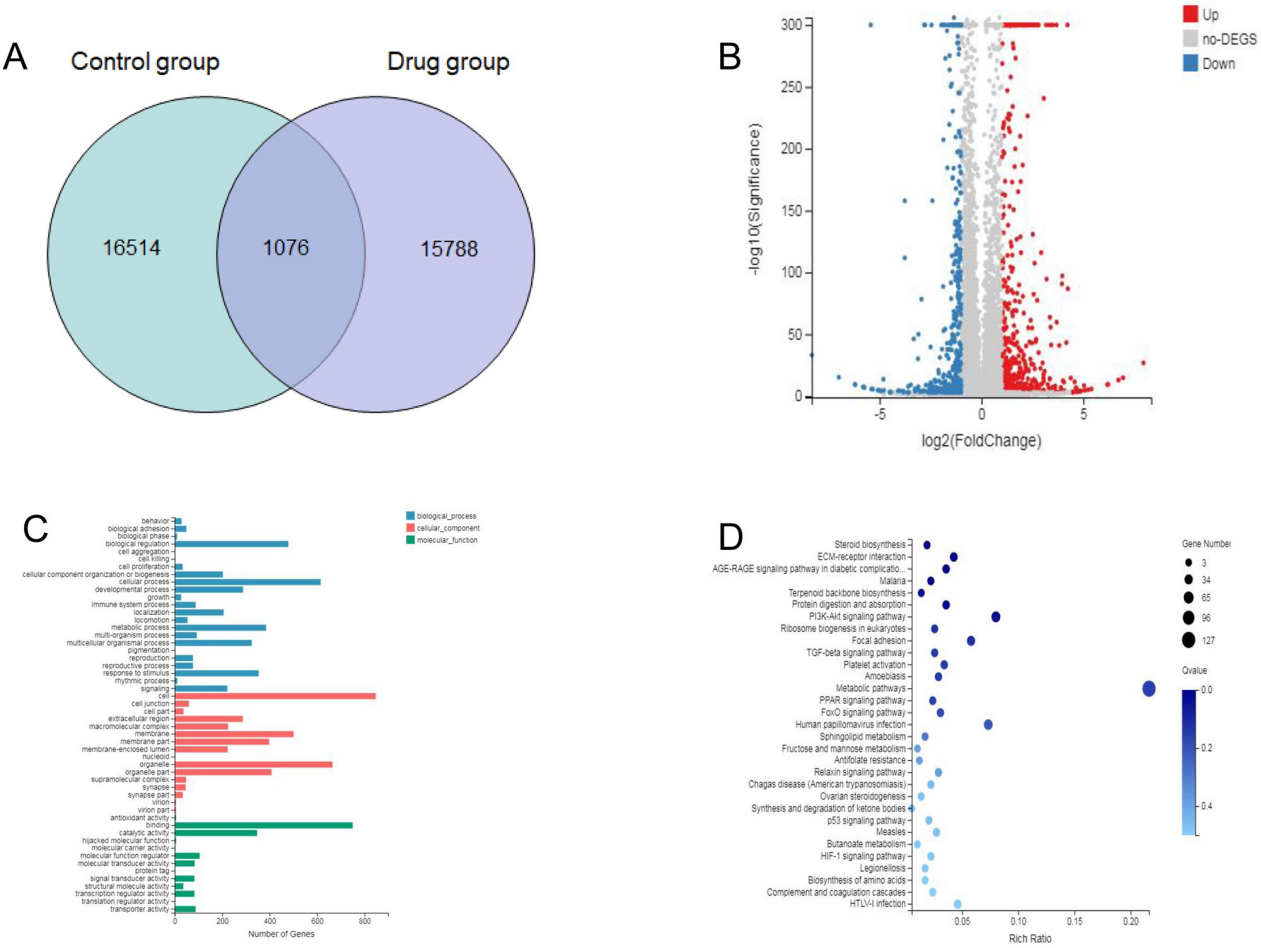
Comparative transcriptomic study of the control and the treatment groups. SGC7901 cells were treated with 1,2,3,6-Tetra-O-galoyl-β-D-glucose at 50 μg/mL for 24 h. **A.** Venn diagram analysis. **B**. Group volcano map. **C.** Those DEGs were enriched in the metabolic process, signalling or transcription factor activation function in GO terms. **D.** PI3-AKT signalling pathway and P53 signalling pathway in KEGG pathways.

To investigate the molecular mechanisms underlying the facilitation of apoptosis by 1,2,3,6-tetra-O-galloyl-β-D-glucose in SGC7901 cells, a BGI-SEQ 500-based RNA-sequencing strategy was applied to compare the gastric cancer mRNA levels between the control and the treatment group. Three pooled mRNA samples from either the control or the treatment group were extracted, library-constructed and sequenced in either the control or the treatment group. The proportion of bases with sequencing error rate ≤ 0.1% in the control group was 88.18%, 88.48%, and 88.22%. The proportion of the number of bases with sequencing error rate ≤ 0.1% was 88.18%, 87.95%, and 88.04% in the 1,2,3,6-tetragalloyl-β-D-glucose-treated group. The total clean reads obtained in each sample were at least 21Mb, and more than 80% of the clean reads were aligned against the human genome database (Table 4). As shown in Venn diagram, a total of 18,038 genes were detected in SGC7901 cells and 1,076 genes were differential expression between the control group and the treatment group (Fig 6A). It can be seen from the gene differential expression analysis of the volcano plot that 563 genes were up-regulated and 513 genes were down-regulated after treatment administration (Fig 6B, S1 Table).

**Table 4:** Summary of data quality assessment.

Sample	Total Raw Reads (M)	Total Clean Reads (M)	Total Clean Bases (Gb)	Clean Reads Q20 (%)	Clean Reads Q30 (%)	Clean Reads Ratio (%)
control_1	21.88	21.72	1.09	96.67	88.18	99.26
control_2	21.88	21.74	1.09	96.76	88.48	99.34
control_3	21.88	21.75	1.09	96.66	88.22	99.39
treat_1	21.89	21.77	1.09	96.72	88.18	99.47
treat_2	21.89	21.75	1.09	96.56	87.95	99.37
treat_3	21.89	21.73	1.09	96.65	88.04	99.27

Note: Clean reads: the total number of reads obtained from filtration, GC%, the percentage of the number of G+C in total number of reads. Q20: the ratio of reads with the sequencing error rate ≤ 1%. Q30: the ratio of reads with the sequencing error rate ≤ 0.1%.

**Fig 7:**
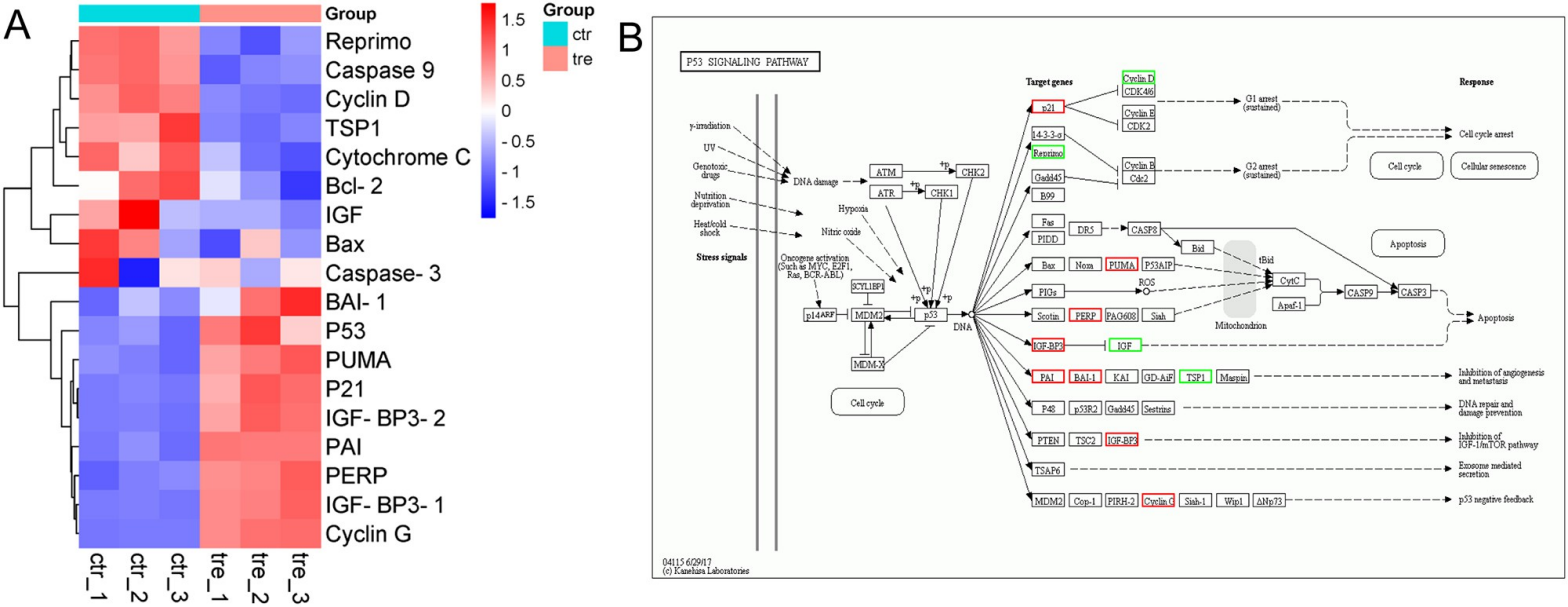
Differential expression analysis of key genes of apoptosis pathway in transcriptome sequencing. A. Heatmap Analysis of Differentially Expressed Genes. **B.** Gene expression changes in P53 signalling pathway.

To highlight the biological processes and molecular functions relevant to DEGs between the two groups, gene ontology (GO) annotation was applied. In the biological process category, 691 DEGs were mapped to a reference list and classified in terms such as RNA metabolic process, tissue development and location (Fig 6C, S2 Table). In the molecular function category, 681 DEGs were mapped and enriched in binding-related terms such as histone binding and transcription regulatory region sequence-specific DNA binding (Fig 6C). The DEG-associated pathways were explored using KEGG pathway enrichment; The top 20 signaling pathways with the most significant differences are listed (Table 5). 1,2,3,6-Tetragalloyl-β-D-glucose may have an effect on SGC7901 cells in terms of energy metabolism, signal transduction and so on. DEGs were mainly enriched in signal transduction pathways such as the PI3-AKT signalling pathway and the P53 signalling pathway (Fig 6D, S3 Table). The differential genes shared by p53 signaling pathway and PI3K-Akt signaling pathway were p21, IGF1, CCND1, IGFBP3 (Table 6). These several genes are closely related to cell proliferation and apoptosis. P21 and CCND1 are cell cycle-related genes, and changes in these two genes can have an effect on the cell cycle. IGFBP3 and IGF1 were associated with apoptosis. P21, IGFBP3 were upregulated and IGF1, CCND1 were downregulated compared with the control group. In addition to the above differential genes in P53 signaling pathway, there were significant differences in PUMA and PERP genes. Both PUMA and PERP were downstream effectors of P53 and could induce apoptosis. The differentially expressed genes in the p53 signaling pathway were shown in the heat map analysis (Fig 7A). In the P53 pathway, the expression levels of several key genes were elevated after 1,2,3,6-tetra-O-galloyl-β-D-glucose treatment, including PUMA, P21, CyclinD and IGF-BP3 (Fig 7B).

**Table 5:** Differential gene KEGG enrichment.

Pathway Name	Gene Num	Rich Ratio	P value
Metabolic pathways	127	0.22	0.006
PI3K-Akt signaling pathway	47	0.08	0.001
Human papillomavirus infection	43	0.07	0.01
Focal adhesion	34	0.06	0.003
ECM-receptor interaction	25	0.04	0
AGE-RAGE signaling pathway in diabetic complications	21	0.04	0
Protein digestion and absorption	21	0.04	0
Ribosome biogenesis in eukaryotes	15	0.03	0.003
TGF-beta signaling pathway	15	0.03	0.005
Platelet activation	20	0.03	0.005
Amoebiasis	17	0.03	0.005
FoxO signaling pathway	18	0.03	0.008
Relaxin signaling pathway	17	0.03	0.024
Measles	16	0.03	0.036
Malaria	13	0.02	0
PPAR signaling pathway	14	0.02	0.007
Chagas disease (American trypanosomiasis)	13	0.02	0.029
P53 signaling pathway	12	0.02	0.035
HIF-1 signaling pathway	13	0.02	0.041

**Table 6:** Differential genes common to p53 signaling pathway and PI3K-Akt signaling pathway.

Pathway	Gene name	description	Fold Difference
P53 signaling pathway, PI3K-Akt signaling pathway	IGFBP3	insulin-like growth factor-binding protein-3	2.36
	CDKN1A, P21, CIP1	Cyclin-dependent kinase inhibitor 1A	1.17
	IGF1	Insulin-like growth factor type-1	-1.41
	CCND1	G1/S specific cyclin-D1	-1.48

**Fig 8:**
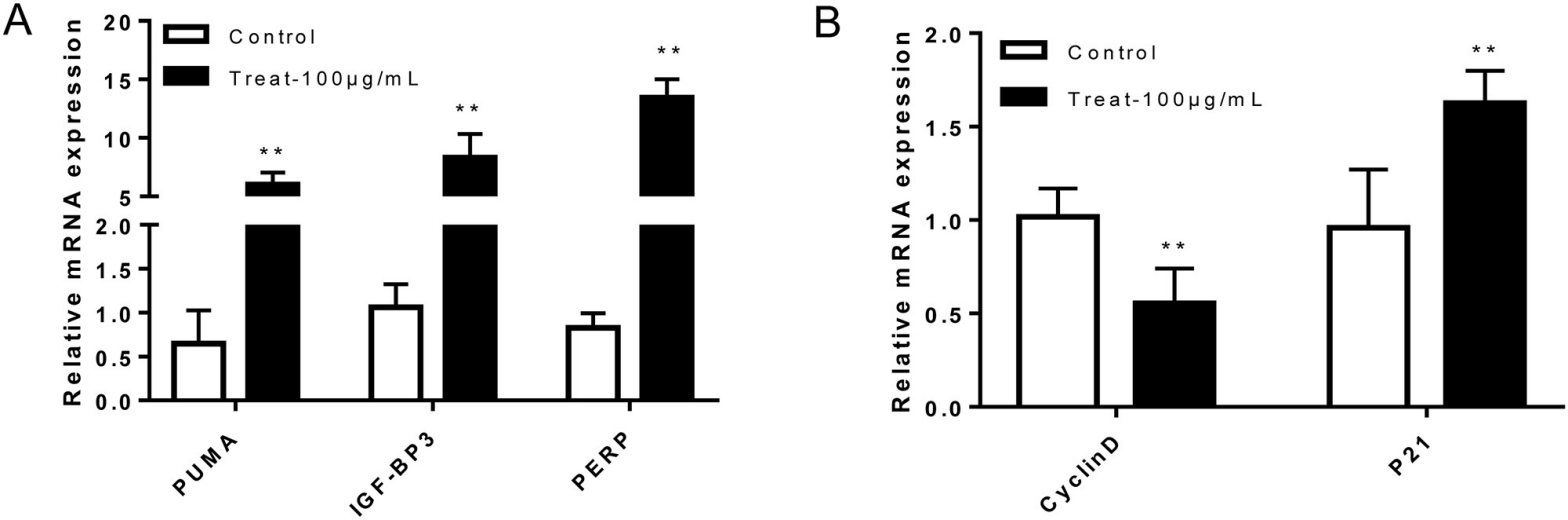
Results of gene detection in SGC7901 cells. qPCR was used to detect changes in mRNA levels of apoptosis-related gene expression in SGC7901 cells. (**A**. *PUMA*, *PERP*, *IGF-BP3*. **B**. *P21*, *CyclinD* Cells were treated with 1,2,3,6-Tetra-O-galoyl-β-D-glucose at 100 μg/mL for 24 h.

We applied 100 μg/mL of 1,2,3,6-tetra-O-galloyl-β-D-glucose to SGC7901 cells for 24 h, and subsequently, qPCR was used to detect changes in mRNA levels of apoptosis-related genes (*P21*, *PUMA*, *PERP*, *IGF-BP3*, *CyclinD*) in the P53 signalling pathway. The results are shown in Fig 8. Compared with the control group, the mRNA expression levels of *P21*, *PUMA*, *PERP* and *IGF-BP3* significantly increased (*P* < 0.01). The expression level of *CyclinD* mRNA was significantly lower than that of the control group (*P* < 0.01). The results show that l,2,3,6-tetra-O-galloyl-β-D-glucose induced different changes in the expression level of apoptosis-related gene mRNA in SGC7901 cells.

### 3.7 Western blot analysis of protein expression treated with 1,2,3,6-tetra-O-galloyl-β-D-glucose

**Fig 9:**
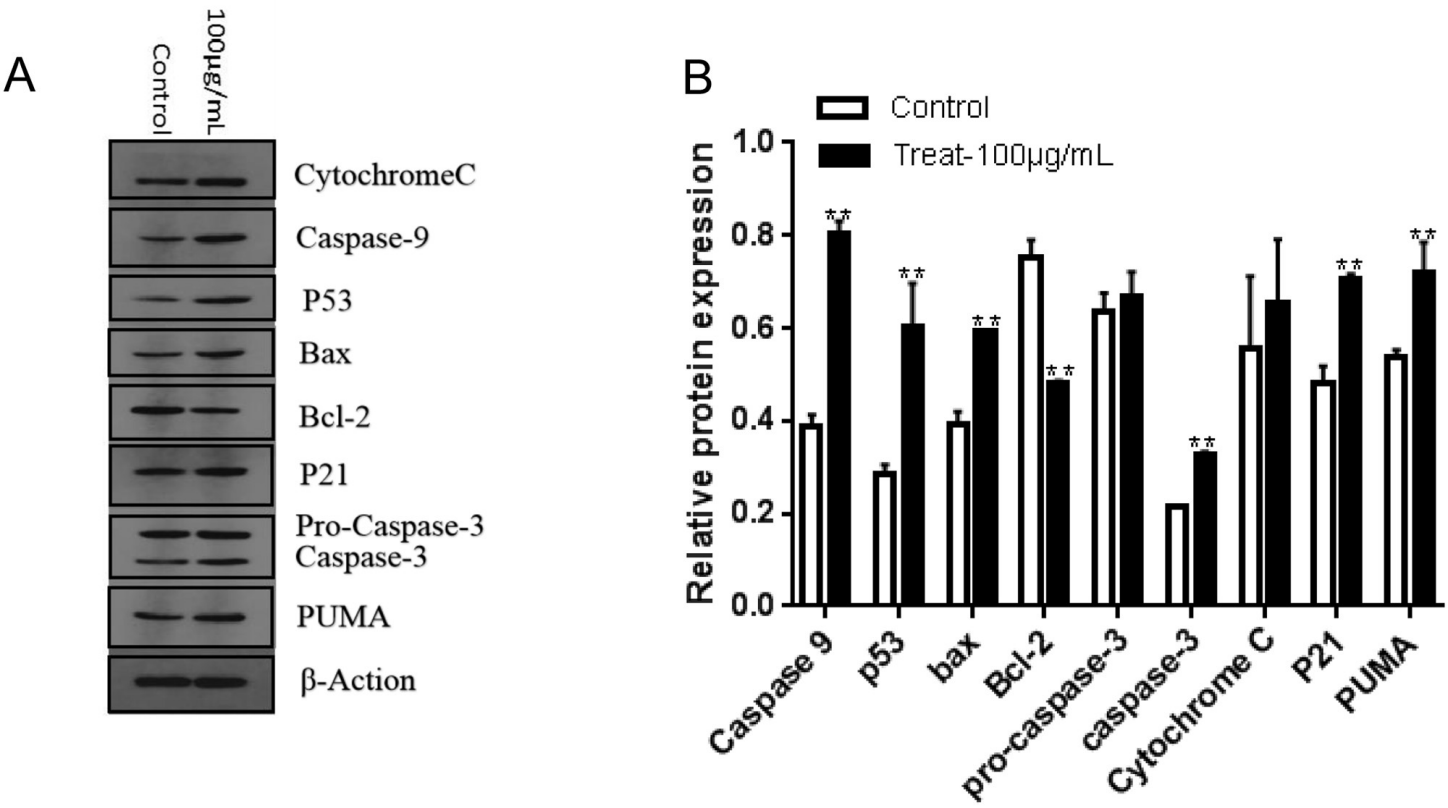
Assessment of Western blotting. A. Western blotting of cytochrome C, P53, Bax, Bcl-2, P21, PUMA, caspase-9, pro-caspase-3 and caspase-3 expression in SGC7901 cells. Cells were treated with 1,2,3,6-Tetra-O-galoyl-β-D-glucose at 100 μg/mL for 24 h. B. The average of protein band grayscale.

To elucidate the mechanism of inducing apoptosis of SGC7901 cells by l,2,3,6-tetra-O-galloyl-β-D-glucose, the expression of P53 and downstream-related proteins was detected by Western blot; the results are shown in Fig 9. After 24 hours of treating SGC7901 cells with 1,2,3,6-tetragalloyl-β-D-glucose, caspase-3 protein was cleaved and its corresponding splicing content increased. The content of caspase-9 also significantly increased (*P* < 0.01), suggesting that caspase protein plays a role in the apoptosis of SGC7901 cells induced by 1,2,3,6-tetragalloyl-β-D-glucose.

Western blot was used to detect changes in the expression levels of Bcl-2 family-related proteins. The results show that the pro-apoptotic protein Bax expression level was up-regulated, and the anti-apoptotic Bcl-2 protein level was significantly decreased (*P* < 0.01). The trend of the Bax/Bcl-2 ratio indicates that Bcl-2 family proteins are involved in the regulation of apoptosis of SGC7901 cells induced by 1,2,3,6-tetra-O-galloyl-β-D-glucose.

Western blot analysis showed that the expression levels of P53, P21 and PUMA were significantly increased compared with the control group (*P* < 0.01), indicating that *P53*, *P21* and PUMA may be involved in the regulation of apoptosis of SGC7901 cells induced by l,2,3,6-tetra-O-galloyl-β-D-glucose.

## 4. Discussion

The mechanism underlying the effect of l,2,3,6-tetra-O-galloyl-β-D-glucose on SGC7901 cells was investigated by transcriptome sequencing. Transcriptomics has been of the most active areas of functional genomics research in the past 2 years [36–38]. After drug intervention in the body or in cells, high-throughput sequencing and screening for changes in related transcriptomes help to understand the targets and possible effects of drug action mechanisms. In this study, RNA-seq detection showed significant changes in the expression of 1,076 genes, of which 513 genes were significantly down-regulated (*P* < 0.01) and 563 genes were significantly up-regulated (*P* < 0.01) in SGC7901 cells treated with 50 μg/mL l,2,3,6-tetra-O-galloyl-β-D-glucose for 48 h. We speculate that these genes mainly affect the proliferation and apoptosis of gastric cancer cells through the P53 signalling pathway.

The ATM belongs to the PIKK family, which could sense DNA damage, transmit DNA damage signals to downstream target proteins and initiate stress systems through cascade amplification, resulting in cycle arrest, repair and even apoptosis [39]. The Chk2 is a substrate for ATM [40], and after DNA damage, ATM allows Chk2 to acquire kinase activity, thereby phosphorylating downstream substrates (such as MDM2, P53 and Cdc25). The P21 is a downstream target protein of P53, belonging to the Chip and Kip families, and a tumour suppressor protein [41]. It is located on the short arm of chromosome 6 (6P21.2); its DNA is 8.6 × 104 bp in length and has three exons. It is a member of the family of cyclin-dependent kinase inhibitors (CDKIs) and the negative regulator of the cycle. The CDKIs, through binding to cyclins, cyclin-dependent kinases (CDKs) or cyclin-CDK, cause cell cycle arrest, thereby blocking cell proliferation. The Insulin-like growth factor binding protein 3 (IGF-BP3) is also a downstream target protein of P53 and promotes apoptosis through IGF-dependent pathways in different tumours [42]. The PERP is a newly discovered P53 downstream effector molecule. In mouse embryonic fibrosis (MEF), PERP is directly regulated by P53 to perform functions in P53-dependent apoptosis, but not in the apoptosis of other pathways [43]. The PUMA gene has first been reported as a P53 transcriptional target gene in 2001 [44]. It is a tumour suppressor gene that induces apoptosis in cancer cells and increases the sensitivity of tumour cells to anti-tumour drugs; PUMA is regulated by the P53 gene, which acts as a transcription factor to induce PUMA expression, and induces apoptosis mainly through the mitochondrial pathway [45]. Similar to Bax, PUMA belongs to the Bcl-2 family of proteins and contains the BH3 domain, which plays a pro-apoptotic role by antagonising the anti-apoptotic protein Bcl-2 [46]. Studies have shown that 1,2,3,6-tetragalloyl-β-D-glucose can increase the mRNA expression of *P21*, *PUMA*, *PERP* and *IGF-BP3* in SGC7901 cells, *CyclinD*. In our study, the mRNA expression significantly decreased (*P* < 0.01).

The mitochondrial apoptotic pathway is a classical apoptotic pathway [47–49]. In the present study, l,2,3,6-tetra-O-galloyl-β-D-glucose caused a decrease in the mitochondrial membrane potential in gastric cancer SGC7901 cells. Western blot showed an increase in the cytochrome c content in the cytosol, indicating that l,2,3,6-tetragalloyl-β-D-glucose promotes the translocation of cytochrome c from the mitochondria into the cytosol. Cytochrome c enters the cytoplasm and activates caspase-9 with the help of Apaf-1; the activated caspase-9 can cleave and activate caspase-3, which is an important apoptosis-executing protein in the apoptotic pathway A variety of apoptosis-related proteins, such as activated caspase-3, ultimately induce apoptosis. In this study, after treatment of gastric cancer cells with l,2,3,6-tetra-O-galloyl-β-D-glucose for 24 h, the expression of caspase-9 in the cytosol was increased and caspase-3 was cleaved, indicating that the cell mitochondrial apoptosis pathway was activated.

We also found that l,2,3,6-tetra-O-galloyl-β-D-glucose can affect the regulatory proteins of the mitochondrial apoptosis pathway. The protein Bcl-2 is a negative regulatory protein in the apoptotic pathway and blocks the formation of dimers of Bax and Bak to inhibit the release of Cytochrome c [49]. In our study, l,2,3,6-tetra-O-galloyl-β-D-glucose down-regulated the expression of Bcl-2; moreover, l,2,3,6-tetra-O-galloyl-β-D-glucose can up-regulate the expression of P53. However, P53 can react with Bcl-2 type polymer, thereby attenuating the inhibitory effect of Bcl-2 on the formation of Bax and Bak dimers.

**Fig 10:**
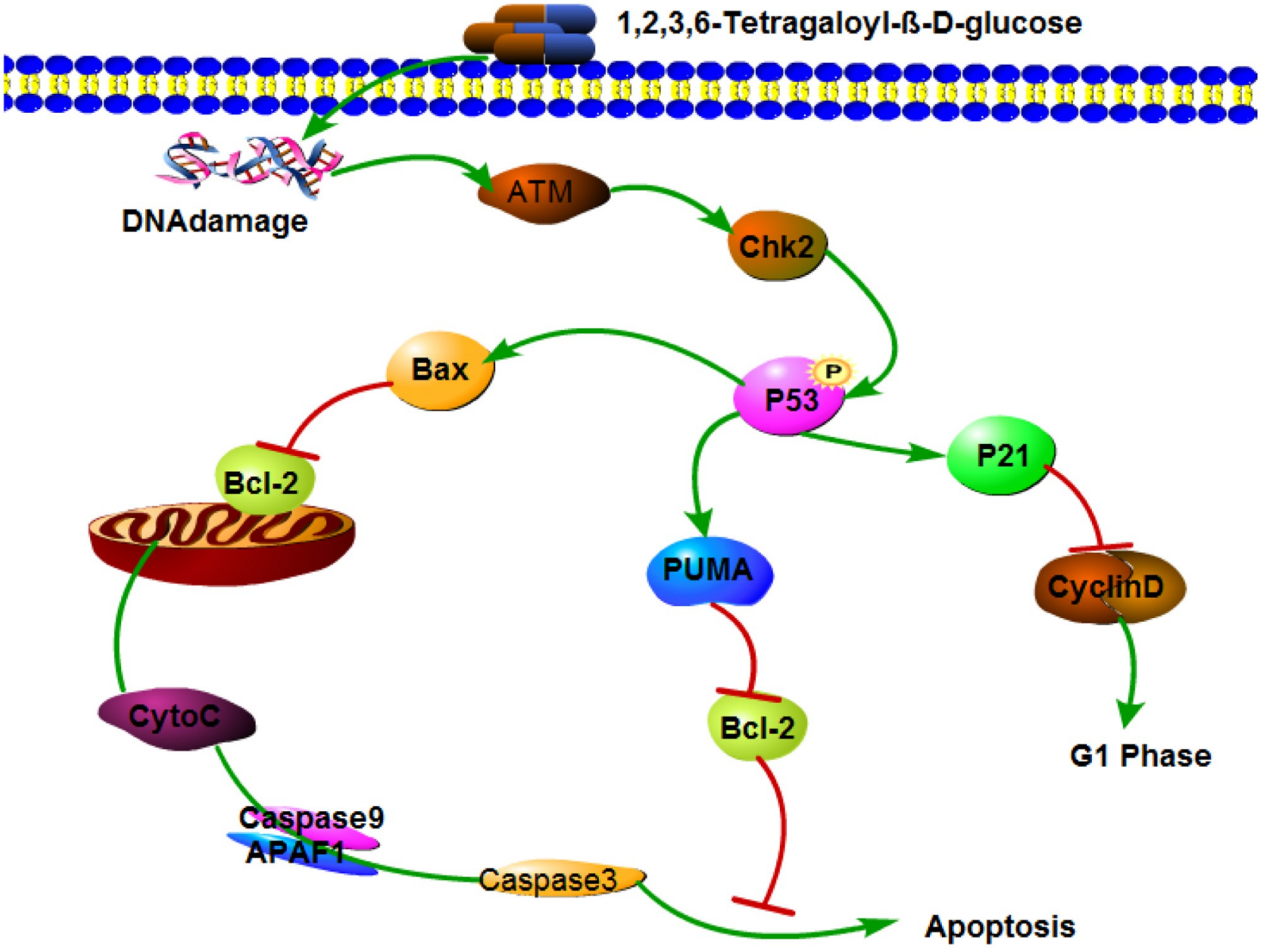
1,2,3,6-Tetra-O-galoyl-β-D-glucose induced apoptosis signalling pathway in SGC7901 cells. l,2,3,6-tetragalloyl-β-D-glucose is effective against human gastric cancer SGC7901 cells in vitro by inhibiting the cell cycle and inducing apoptosis in tumour cells.

In summary, l,2,3,6-tetra-O-galloyl-β-D-glucose has an effect against human gastric cancer SGC7901 cells *in vitro*. One of the mechanisms is the inhibition of the cell cycle; l,2,3,6-tetra-O-galloyl-β-D-glucose can up-regulate the expression of the P21 gene and down-regulate the expression of the CyclinD gene to arrest SGC7901 cells in the G1 phase. Another mechanism is the induction of apoptosis in tumour cells. In SGC7901 cells, the mitochondrial membrane potential decreased and the intracellular calcium ion concentration increased under the action of 1,2,3,6-tetra-O-galloyl-β-D-glucose. Cytochrome c is transferred from the mitochondria to the cytoplasm, and the mitochondrial apoptosis pathway-associated proteins caspase-9 and caspase-3 are activated. At the same time, l,2,3,6-tetra-O-galloyl-β-D-glucose also up-regulates P53 protein expression, activates the P53 signalling pathway, weakens the Bcl-2 inhibition of Bax and promotes cytochrome c release to induce apoptosis of SGC7901 cells (Fig 10).

## 5. Conclusions

We isolated 1,2,3,6-tetra-O-galloyl-β-D-glucose from an ethanol extract of TbR shells. This substance has a good inhibitory effect on SGC7901 cells and could affect the cell cycle of SGC7901, arrest cells in the G1 phase, induce apoptosis in SGC7901 cells, increase the intracellular calcium concentration and decrease the mitochondrial membrane potential. Transcriptome sequencing analysis showed that 1,2,3,6-tetra-O-galloyl-β-D-glucose may induce apoptosis in SGC7901 cells via the P53 signalling pathway. The results of qPCR and Western blot show that 1,2,3,6-tetra-O-galloyl-β-D-glucose could up-regulate the expression of the *CyclinD* gene by up-regulating the expression levels of *P21*, *PUMA*, *PERP* and *IGF-BP3* genes. It also increased the expression levels of P53, Bax, cytochrome C, caspase-3 and caspase-9 and decreased that of Bcl-2, thereby inducing apoptosis of SGC7901 cells.

## Supporting information

S1 Table The expression of DEGs in treat-vs-control group. (XLSX)

S2 Table Distribution of DEGs in different GO terms. (XLSX)

S3 Table The KEGG enrichment of DEGs. (XLSX)
